# Cerebral Oxygenation Responses to Standing in Young Patients with Vasovagal Syncope

**DOI:** 10.3390/jcm12134202

**Published:** 2023-06-21

**Authors:** Laura Pérez-Denia, Paul Claffey, Ailbhe O’Reilly, Maria Delgado-Ortet, Ciara Rice, Rose Anne Kenny, Ciarán Finucane

**Affiliations:** 1 School of Medicine, Trinity College Dublin, D02 K6K6 Dublin, Ireland; 2 Mercer’s Institute for Successful Ageing, St. James’s Hospital Dublin, D08 TYF3 Dublin, Ireland; 3 Department of Medical Physics, Mercer’s Institute for Successful Ageing, St. James’s Hospital Dublin, D08 C9X2 Dublin, Ireland; 4 Department of Bioengineering, School of Mechanical Engineering, Trinity College Dublin, D02 PN40 Dublin, Ireland; 5 Department of Radiology, University of Cambridge, Cambridge CB2 0QQ, UK

**Keywords:** syncope, cerebral oxygenation, standing, near-infrared spectroscopy, orthostatic, young

## Abstract

Vasovagal syncope (VVS) is common in young adults and is attributed to cerebral hypoperfusion. However, during active stand (AS) testing, only peripheral and not cerebral hemodynamic responses are measured. We sought to determine whether cerebral oxygenation responses to an AS test were altered in young VVS patients when compared to the young healthy controls. A sample of young healthy adults and consecutive VVS patients attending a Falls and Syncope unit was recruited. Continuous beat-to-beat blood pressure (BP), heart rate, near-infrared spectroscopy (NIRS)-derived tissue saturation index (TSI), and changes in concentration of oxygenated/deoxygenated Δ[O_2_Hb]/Δ[HHb] hemoglobin were measured. BP and NIRS-derived features included nadir, peak, overshoot, trough, recovery rate, normalized recovery rate, and steady-state. Multivariate linear regression was used to adjust for confounders and BP. In total, 13 controls and 27 VVS patients were recruited. While no significant differences were observed in the TSI and Δ[O_2_Hb], there was a significantly smaller Δ[HHb] peak-to-trough and faster Δ[HHb] recovery rate in VVS patients, independent of BP. A higher BP steady-state was observed in patients but did not remain significant after multiple comparison correction. Young VVS patients demonstrated a similar cerebral circulatory response with signs of altered peripheral circulation with respect to the controls, potentially due to a hyper-reactive autonomic nervous system. This study sets the grounds for future investigations to understand the role of cerebral regulation during standing in VVS.

## 1. Introduction

Syncope is defined as a transient loss of consciousness due to cerebral hypoperfusion that resolves in a rapid and spontaneous manner [1], with vasovagal syncope (VVS) being its most common form in teenagers and young adults, who rarely experience cardiac syncope [2]. Exaggerated orthostatic cardiovascular responses have been recently observed in patients with VVS, a behavior that has been attributed to a hyper-reactive autonomic nervous system, and hypothesized to bring the body closer to its physiological limit, increasing an individual’s susceptibility to VVS during episodes of hypotension [3,4].

The European Society of Cardiology recommends that patients investigated for VVS undergo the active stand (AS) test with concurrent peripheral hemodynamics monitoring to identify potential syncope-related conditions such as orthostatic hypotension (OH), delayed blood pressure recovery, or postural orthostatic tachycardia syndrome (POTS) [1,5,6].

In complex VVS cases, a head-up tilt (HUT) test is performed [1]. Two recent studies from our group have demonstrated that VVS diagnosis can be predicted from AS cardiovascular responses [5,6]. Despite the obvious differences in these test protocols (AS and HUT), these studies reveal an overlap in the physiological mechanisms that they trigger, suggesting that the AS test might be sufficient to unveil abnormalities in such mechanisms and might assist more in the diagnosis of VVS than currently considered. 

In standard practice, cerebral circulation is not directly assessed during the AS and the HUT tests, and only inferred from peripheral signals and orthostatic intolerance symptoms, often being poor surrogates [7,8]. Near-infrared spectroscopy (NIRS) has emerged as a potential tool for the clinical evaluation of syncope [9,10,11], providing measures of cerebral tissue oxygenation. While cerebral oxygenation responses to a HUT test have been characterized in VVS patients, scarce data can be found on the responses to an AS test, with available literature focusing on healthy young individuals [12,13,14], patients with autonomic failure [15] and orthostatic intolerance [16,17], and children with OH, delayed OH, POTS, and VVS [18,19,20]. Despite the crucial role of the AS test in VVS assessment and the increasing interest in the use of NIRS in this context, an important gap can be identified: cerebral oxygenation responses to an AS are yet to be investigated in young patients with VVS. 

The aim of this study is to investigate the following research questions: Are there differences in cerebral oxygenation and peripheral hemodynamics measured during an AS test, between young patients with VVS and the healthy controls? Based on previous research [5,6], we hypothesize that VVS patients will have an exaggerated peripheral hemodynamics response to an AS test. Similarly, we hypothesize that a distinctive cerebral oxygenation response will be found in VVS patients as compared to the healthy controls. Differences in the peripheral and cerebral responses to an AS test might represent important biomarkers to identify patients with VVS, further our understanding of their pathophysiology, and aid in their assessment and management. 

## 2. Materials and Methods

### 2.1. Setting and Participants 

Consecutive patients were recruited prospectively from a National Falls and Syncope Unit at St James’s Hospital, Dublin, Ireland. Patients were referred for investigation of suspected syncope or presyncope and had a history of presyncope and/or syncope. 

A sample of young healthy community-dwelling controls was also prospectively recruited. The study had ethical approval from the Tallaght University Hospital/St. James’s Hospital Joint Research Ethics Committee, and informed consent was written and signed by all participants.

### 2.2. Inclusion and Exclusion Criteria 

Patients and controls were aged 18–30 years. Patients had a clinical diagnosis of VVS, determined by a clinical expert in syncope. Controls with a history of syncope, cardiovascular disease, or other comorbidities were excluded. All controls were physically fit and were not taking any cardioactive medication. Participants were excluded if they were not able to stand up from a lying position and stay standing for at least 2 min. Women self-reporting pregnancy were excluded. 

### 2.3. Active Stand Testing

#### 2.3.1. Protocol

Participants underwent an AS test as per international guidelines [1,21]. Participants lay supine for 5–10 min until hemodynamic stabilization and were then instructed to stand up as quickly as possible and remain standing quietly for 3 min. Assistance was provided during the stand if necessary. 

#### 2.3.2. Equipment

During AS testing, frontal lobe cerebral oxygenation was monitored using NIRS (Portalite, Artinis Medical Systems B.V., Elst, The Netherlands) to derive the values of the tissue saturation index (TSI) (units of percentage (%)) and changes in the concentration of oxyhemoglobin (Δ[O_2_Hb]) and deoxyhemoglobin (Δ[HHb]) (in µmol/L). The sensor was placed on the left side of the forehead, centered at 3 cm lateral and 3.5 cm above the nasion [22]. An opaque head bandage was applied to provide ambient light protection and minimize sensor motion artifact. 

Continuous beat-to-beat blood pressure (BP) (systolic BP (SBP) and diastolic BP (DBP)) and HR were also measured using a Finometer NOVA (Finometer NOVA, Finapres Medical Systems, Amsterdam, The Netherlands). A finger cuff was attached to the middle phalanx of a finger of the left hand, and a height correction unit was applied to correct hydrostatic effects. The Finometer signal was further calibrated using Physiocal™ and brachial calibration based on the average of two oscillometric BP measurements recorded during the resting period. Physiocal™ was turned off on standing to avoid data loss. 

#### 2.3.3. Cohort Clinical Characteristics

A comprehensive medical history was recorded and included the following information: anthropometrics (age (years), gender, weight (kg), height (m), cardiovascular diseases, neurological diseases, vestibular disorders, psychological disorders, vertigo and vestibular disorders, obstructive sleep apnea, chronic obstructive airway disease, and endocrine diseases. Behavioral health including smoking status (non-smoker, current smoker, or ex-smoker) and history of excess alcohol intake (>14 units/weeks for women, >21 units/weeks for men) was also recorded. A detailed medication record was also taken at the time of testing.

### 2.4. Data Analysis

#### 2.4.1. Signal Processing

**Figure 1:**
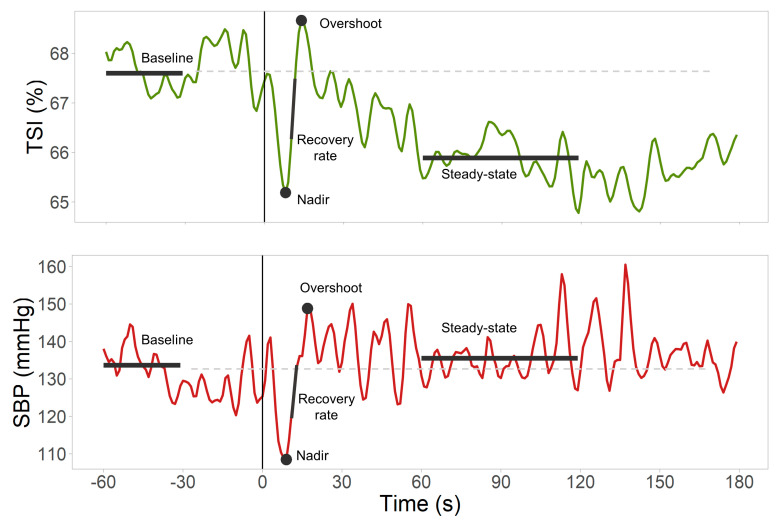
Features extracted from TSI and SBP responses to an active stand test in a selected participant. Features extracted from the Finometer and NIRS signals (i.e., baseline, nadir, recovery rate, overshoot, and steady—state) are indicated in both graphs. The standing moment is indicated by a black vertical line at t = 0. The baseline value is indicated by a gray dashed line across the graph. SBP = systolic blood pressure, TSI = tissue saturation index.

Custom code was developed in MATLAB R2020a (The Math Works, Inc., MATLAB, Version 2020a, Natick, MA, USA) to process the multimodal physiological data collected as previously described [23,24]. The following biosignals were analyzed: SBP, DBP, HR and TSI, Δ[O_2_Hb], and Δ[HHb], with a standardized process applied to all biosignals, described in detail in our recent article [25]. A description of the features extracted is detailed in Table 1 and depicted in Figure 1 (Table A1 for an expanded version of features definition). Features extracted included baseline, nadir (or peak in case of Δ[HHb], as it presents an inverted morphology), overshoot (or trough in the case of Δ[HHb]), maximum recovery rate between nadir and overshoot, and steady-state. In NIRS-derived signals (TSI/Δ[O_2_Hb]/Δ[HHb]), a recovery rate normalized by SBP changes was also derived. 

Duration of supine rest was estimated from the duration of the Finometer recording prior to standing. Speed of standing was obtained by processing the height correction signal to identify the start and stop of the standing maneuver [23].

OH was defined as a sustained (for the duration of the stand) drop from baseline larger than 20 mmHg in SBP and/or a drop larger than 10 mmHg DBP. 

#### 2.4.2. Statistical Analysis

Statistical analysis was performed using Rstudio (RStudio Team, 2016, Rstudio: Integrated Development for R. Rstudio, Inc., Boston, MA, USA). All data were assessed for normality using histograms, Q-Q plots, and the Shapiro–Wilk test. Differences in baseline characteristics between healthy participants and patients were assessed using a Mann–Whitney U test to account for the relatively small number of subjects in the study and deviations from normality. A Chi-squared test was used for binary variables. Values are reported as median and interquartile range (IQR). Linear regression was employed to evaluate unadjusted differences in SBP and cerebral oxygenation (Model 1) features between the two groups. Multivariate linear regression was used to adjust for a number of covariates as part of three adjusted models, described in Table 2. Outliers were identified by inspection for abnormal values, incomplete and noisy data, and/or standardized residual greater than 3 [26] Based on this, four outliers were identified, and after sensitivity analyses they were removed from the final analysis. A false discovery rate (FDR) procedure was used to correct for multiple testing comparisons [27]. Differences were considered to be statistically significant for *p*-value < 0.05.

Post hoc sensitivity analyses were performed to assess the robustness of the above results to the following factors: (1) adjustment for the effects of standing speed and duration of lying; (2) definition of recovery rate and normalized recovery rate as averaged rates between nadir and overshoot rather than local maxima rates, (3) age and gender matching of a subset of patients to the group of the controls. 

## 3. Results

### 3.1. Patients’ Characteristics

A total of 40 participants were recruited: 27 patients and 13 controls. A summary of the participants characteristics is presented in Table 3. Participants did not present significant differences in demographic and baseline hemodynamic characteristics, except for the baseline HR, which was significantly higher in VVS patients (76 (14) vs. 66 (16), *p* = 0.039). There was a higher proportion of women in the patient vs. the control group (78% vs. 54%, *p* = 0.122). VVS patients presented with different comorbidities (cardiovascular diseases, cholesterol, chronic obstructive airway disease, migraine, depression/anxiety) with eight taking medications. The controls did not have any comorbidities. All patients had either a history of syncope or presyncope in the last year, with 59% and 41% having a history of recurrent syncope and presyncope, respectively. 

### 3.2. Univariate Analyses 

**Figure 2:**
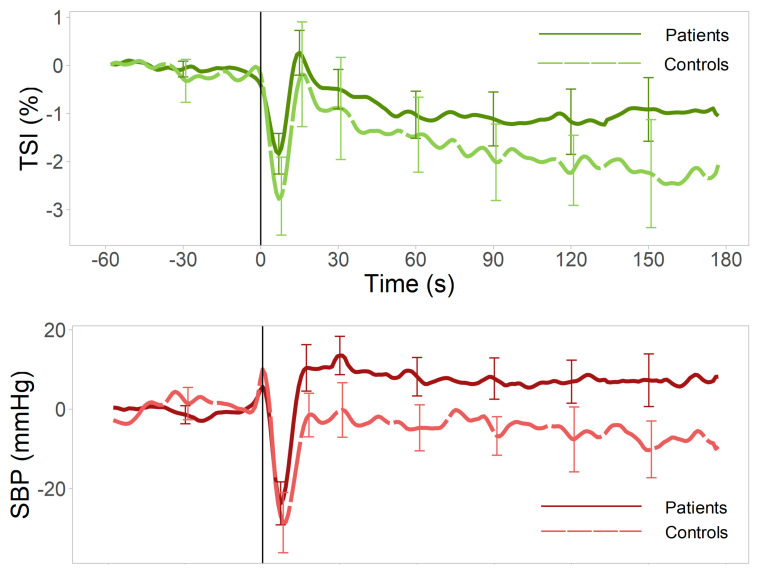
Average TSI and SBP responses for VVS patients and controls. Average (5% trimmed mean and interquartile range) traces for SBP (mmHg) and TSI (%) relative to baseline for VVS patients (dark color, solid line) and controls (light color, dashed line). Stand occurred at t = 0, indicated by a black vertical line. VVS patients presented a higher SBP value during steady—state (average value during 60–120 s) and smaller initial drop after standing than controls. SBP = systolic blood pressure, TSI = tissue saturation index, VVS = vasovagal syncope.

**Figure 3:**
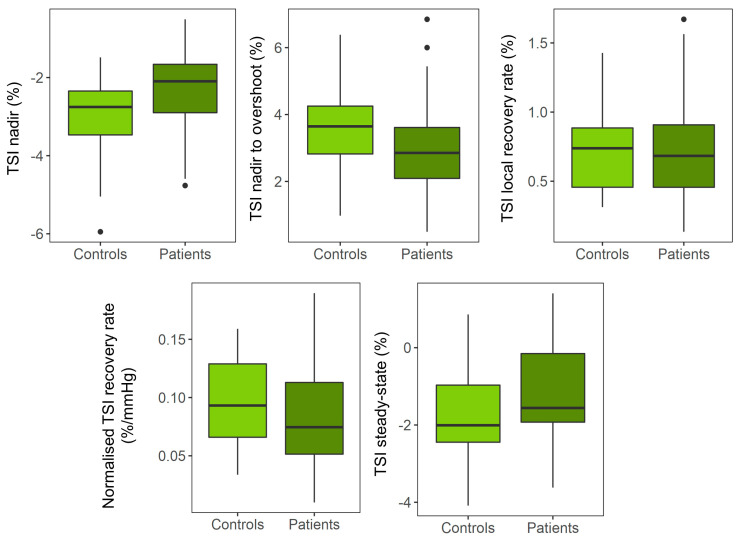
Differences in TSI between patients and controls for the TSI features analyzed. TSI nadir, TSI nadir to overshoot, maximum TSI recovery rate, normalized TSI recovery rate, and TSI nadir steady—state. None of the differences were significant after correction for multiple testing (see Table 4). TSI = tissue saturation index.

Ensemble average responses are depicted in Figure 2 for both patients and the controls. Analyses revealed a significantly smaller TSI nadir (β: 0.93, CI: [0.12 1.74], *p* = 0.026) (Table 4, Figure 3) and Δ[O_2_Hb] nadir (β: 3.62, CI: [0.05 7.19], *p* = 0.047) (Table A2), which were not significant after FDR correction. VVS patients had an attenuated Δ[HHb] response, both in terms of the peak magnitude (β: −1.09, CI: [2.07 −0.12], *p* = 0.019) and recovery rate (β: 0.29, CI: [0.09 0.48], *p* = 0.005) (Table A3). Patients recovered to a higher SBP steady-state value above baseline when compared to the controls (β: 10.80, CI: [3.42–18.17], *p* = 0.005), which remained significant after correcting for multiple testing (Table A4). 

### 3.3. Multivariate Analyses

A smaller drop was observed in the TSI nadir when correcting for gender, BMI, and baseline HR (Model 3: β: 0.84, CI: [−0.13 1.80], *p* = 0.080) (Table 4). No associations were observed when further adjusting for the concurrent SBP variable in Model 4. A slower Δ[O_2_Hb] recovery rate was also observed in patients (β: −0.37, CI: [−0.72 −0.01], *p* = 0.046), which did not hold significant after the FDR adjustment (Table A2). In the case of Δ[HHb], multivariate analysis yielded similar results to the univariate analysis (Table A3). 

Regarding SBP, a significantly higher steady-state value was present in patients after correcting for gender, BMI, and HR baseline (β: 8.37, CI: [0.44 16.30], *p* = 0.039), which was not significant after the FDR adjustment (Table A4). 

### 3.4. Sensitivity Analyses

Correction for the duration of lying and speed of standing slightly strengthened the TSI association at the nadir (Table A5). Defining the recovery and normalized recovery rates as averaged rates did not impact the associations detected (Table A6). Furthermore, when repeating the analysis with a subset of patients matched with the controls in age and gender, the differences in cerebral oxygenation and SBP were in a similar direction but somewhat reduced (Table A7 and Table A8). 

## 4. Discussion

This study led to the following novel observations: (1) the AS initial cerebral tissue oxygenation response in patients diagnosed with VVS is similar to that of the healthy controls, (2) standing steady-state SBP is higher in VVS patients. 

VVS patients presented with an attenuated initial Δ[HHb] response when compared to the controls. These effects were reduced in the age and gender matched subset comparison. Furthermore, no significant associations were observed in the TSI or Δ[O_2_Hb] responses. To our knowledge, this is the first study that compares AS cerebral oxygenation responses between young VVS patients and the controls. From a group of healthy young adults, Kim et al. observed that those who reported symptoms of orthostatic intolerance during a squat-to-stand test had a slower and more delayed drop rate in total hemoglobin with respect to asymptomatic participants [16]. Lower Δ[O_2_Hb] initial drops and steady-state values were also reported in children with abnormal (e.g., orthostatic hypotension, VVS) peripheral hemodynamic responses to an AS as compared to a control group [19]. The first study has a similar population to ours, although their squat-to-stand-based protocol may have elicited stronger hemodynamic waves [16], potentially explaining the differences with the current study. The second study investigated a heterogeneous population of children with different orthostatic conditions, which poses a challenge when comparing to our study. 

Cerebral oxygenation patterns in patients with VVS have also been studied during the HUT test [9,10,11,28]. These studies have typically demonstrated prolonged lower levels of cerebral oxygenation as measured by NIRS in patients with a positive HUT response when compared to the healthy controls [9,28]. In our study, the primary NIRS changes occurred in the first 30 s after standing while changes during HUT usually occurred over longer windows of time and are at slower rates. These differences are most likely attributed to the differences in the physiological mechanisms triggered by the AS and the HUT, with one involving active muscle contraction and a rapid change in posture and the other one representing a passive and slower orthostatic challenge, respectively.

An alternative method to NIRS to measure cerebral circulation during standing is transcranial doppler (TCD) ultrasonography, which provides a measure of cerebral blood flow velocity (CBFv) in the brain arteries as a surrogate for cerebral blood flow [29]. To the best of the authors’ knowledge, no studies have investigated transient changes in CBFv during active standing in adult patients with VVS. One study performed AS tests in children with syncope symptoms, observing no differences in the orthostatic response between patients and the controls [30]. However, the values were averaged over a 5 min period, and therefore comparison to our study is challenging. Other studies investigating TCD responses to standing in patients with other orthostatic syndromes (POTS and diabetes accompanied by OH) have reported decreases in CBFv associated with symptoms of orthostatic intolerance and decreases in BP [31,32]. Differences in the severity and pathophysiological mechanisms leading to syncope, POTS, and diabetes-related OH might explain the differences with our study. 

The shape of the cerebral perfusion response depends on different factors, including the passive effect of the underlying BP waveform and the active modulation of CA. Differences in the initial Δ[HHb] response observed might indicate differences in brain activation between the two cohorts [33], with patients potentially having a less pronounced activation. However, these differences were not reflected in the TSI, with only the drop at the nadir demonstrating a smaller value in patients univariately (β ~1%). When correcting for covariates, despite the association being no longer significant, its magnitude was still relatively large (β ≥ 0.8%), considering that a 5%–10% drop can cause syncope. Therefore, the current study might motivate future research with larger sample sizes to investigate this potential difference. Nevertheless, the current results do not provide enough evidence to support that the overall cerebral tissue oxygenation (TSI) is affected in VVS patients with respect to the healthy controls.

Interestingly, a higher SBP steady-state was observed in patients. Our group previously reported an exaggerated peripheral hemodynamic response to standing in patients with VVS vs. the controls both in young [6] and older adults [5]. This led to the novel hypothesis that patients with VVS may have a hyper-reactive autonomic nervous system [5,6]. Similarly, recent evidence from Brignole et al. demonstrated that VVS patients presented increased resting HR and DBP [34]. The authors proposed that the decreased venous return and stroke volume observed may have led to chronic compensatory increased sympathetic outflow. Here, higher resting HR and exaggerated BP responses were observed in the VVS patient group vs. the controls. These responses may seem protective during an AS, although they might be a sign of potentially enhanced compensatory mechanisms that bring the neurocardiovascular system closer to its physiological limit, requiring a smaller orthostatic challenge to activate the vasovagal reflex and cause VVS (i.e., hyper-reactivity). The lack of correspondence between cerebral oxygenation and SBP might indicate that cerebral autoregulation (CA) is preserved in patients with VVS, although further research is required to better understand whether CA and other regulators of cerebral oxygenation such as PaCO_2_ could be altered in VVS patients [10]. 

### 4.1. Limitations

Firstly, NIRS interpretation is challenging, as the arterial/venous/capillaries proportion of the NIRS signal is unclear, although venous circulation is believed to be the main contributor [10]. Furthermore, NIRS does not provide a global measure; therefore, other regions of the cerebral circulation are not captured. Extracerebral contamination, differences in tissue morphology captured, thickness of extracerebral layers, and susceptibility to motion artifacts are also methodological limitations to consider in NIRS [35,36,37]. Lastly, CO_2_ is one of the strongest vasoactive molecules that affect the cerebrovasculature and CA [10,38], with PaCO_2_ correlating to cerebral blood flow. Thus, it remains unclear whether the results found could be attributed to changes in PaCO_2_ as a result of postural hyperventilation [39,40]. 

### 4.2. Strengths

An AS was used instead of the commonly utilized head-up tilt test to study a population with syncope, which provides a simpler and faster way of assessing cerebrovascular regulation upon an orthostatic challenge [41]. The presented application of NIRS is novel and provides a wider picture of an individual’s physiology. Correction for BP has also been applied and allowed investigating the extent of its driving effect. Relative changes from the baseline were investigated, a more accurate and reliable measure when compared to the absolute values of NIRS-derived signals. Furthermore, the influence of the duration of lying and speed of standing was examined, which have been previously demonstrated to influence the responses to an AS [23], but did not alter the results obtained. 

## 5. Conclusions

This study furthers our understanding of transient cerebral and peripheral hemodynamics of VVS patients during standing. Our results suggest cerebral oxygenation is similar while BP demonstrates an exaggerated response, supporting previous evidence of a hyper-reactive autonomic nervous system. The incorporation of new emerging technologies such as NIRS in this clinical context can provide a wider integrative view of the patients beyond traditional approaches, help us better understand their physiology, and further personalize their management. 

## Figures and Tables

**Table 1:** Definitions, units, description of variables used in the analyses.

Biosignal (Units)	Variable	Description
SBP (mmHg), HR (bpm), TSI (%)	Baseline	Mean value 30–60 s before standing
SBP (mmHg), TSI (%)	Nadir	Largest trough after standing (in first 30 s)
SBP (mmHg)	Overshoot	First peak found between SBP nadir time +5 s and 60 s after standing
TSI (%)	Overshoot	First peak found between TSI nadir time +2 s and 60 s after standing
SBP (mmHg), TSI (%)	Steady-state	Mean value 60–120 s after standing
SBP (mmHg), TSI (%)	Delta	Nadir—Baseline
SBP (mmHg), TSI (%)	Nadir to overshoot	Overshoot—Nadir
SBP (mmHg), TSI (%)	Steady-state change	Steady-state—Baseline
SBP (mmHg/s), TSI (%/s)	Maximum recovery rate	Largest difference between consecutive points divided by the time between points (within the time interval between the nadir and overshoot)
TSI/SBP (%/mmHg)	Normalized TSI recovery rate	Maximum TSI recovery rate/maximum SBP recovery rate

HR: heart rate (units: beats per minute (bpm)), SBP: systolic blood pressure (units: mmHg); TSI: tissue saturation index (units: %).

**Table 2:** Correction for covariates in SBP, TSI, Δ[O_2_Hb], and Δ[HHb] regression analyses.

	Covariates
Model 1	None
Model 2	Age + Gender
Model 3	Age + Gender + BMI + HR baseline
Model 4	Age + Gender + BMI + HR baseline + SBP concurrent feature *

* Model 4 only applicable for NIRS variables (TSI, Δ[O_2_Hb], and Δ[HHb]): e.g., when the dependent variable was “TSI nadir”, then “SBP nadir” was used as a covariate in the model. BMI: body mass index, SBP: systolic blood pressure, TSI: tissue saturation index, Δ[O_2_Hb]: concentration of oxygenated hemoglobin, Δ[HHb]: concentration of deoxygenated hemoglobin.

**Table 3:** Participants’ Characteristics.

	Controls (n = 13)	Patients (n = 27)	*p*-Value
Age (years)	22 (1)	21 (6)	0.220
Female, % (n)	54 (7)	78 (21)	0.122
Weight (kg)	75 (15)	69 (17)	0.496
Height (cm)	174 (11)	168 (13)	0.310
BMI (kg/m^2^)	23 (3)	23 (5)	0.806
SBP baseline (mmHg)	132 (22)	130 (27)	0.479
DBP baseline (mmHg)	81 (5)	77 (18)	0.875
MAP baseline (mmHg)	101 (11)	102 (23)	0.977
HR baseline (bpm)	66 (16)	76 (14)	0.039 *
Cardiovascular diseases, % (n)	0	4 (1)	-
Cholesterol, % (n)	0	4 (1)	-
Chronic obstructive airway disease, % (n)	0	15 (4)	-
Migraine, % (n)	0	15 (4)	-
Depression/anxiety, % (n)	0	30 (8)	-
Current smokers, % (n)	23 (3)	15 (4)	0.519
Previous smokers, % (n)	0	0	-
History of syncope, % (n)	0	85 (23)	-
History of recurrent syncope, % (n) *	0	59 (16)	-
History of presyncope, % (n)	0	52 (14)	-
History of recurrent presyncope, % (n) *	0	41 (11)	-
Presence of OH on standing	8 (1)	0	-
Beta-blockers, % (n)	8 (1)	4 (1)	-
Antidepressants, % (n)	8 (1)	11 (3)	-
Antihypertensives, % (n)	0	0	-

Values expressed as median (interquartile range). * *p* < 0.05. SBP, DBP, MAP, and HR values were derived from the Finometer measurements, which are calibrated using an oscillometric arm-cuff-based brachial calibration process. Recurrent syncope/presyncope is defined as a history of two or more syncope/presyncope episodes. BMI: body mass index, DBP: diastolic blood pressure, HR: heart rate, MAP: mean arterial pressure, SBP: systolic blood pressure, OH: orthostatic hypotension.

**Table 4:** Univariate (model 1) and multivariate analyses of the effects of group (patients vs. controls (reference)) on TSI responses to active stand testing.

	β Coefficient (CI)	*p*-Value	FDR Corrected *p*-Value
Model 1			
TSI nadir (%)	0.93 (0.12 1.74)	0.026 *	0.130
TSI nadir to overshoot difference (%)	−0.53 (−1.55 0.49)	0.300	0.500
Maximum TSI recovery rate (%/s)	−0.04 (−0.30 0.23)	0.790	0.790
Normalized TSI recovery rate (%/mmHg)	−0.01 (−0.04 0.02)	0.426	0.532
TSI baseline to steady-state change (%)	0.72 (−0.27 1.71)	0.148	0.370
Model 2			
TSI nadir (%)	0.84 (0.00 1.68)	0.050	0.250
TSI nadir to overshoot difference (%)	−0.62 (−1.68 0.44)	0.242	0.318
Maximum TSI recovery rate (%/s)	−0.06 (−0.33 0.22)	0.688	0.688
Normalized TSI recovery rate (%/mmHg)	−0.02 (−0.05 0.01)	0.254	0.318
TSI baseline to steady-state change (%)	0.66 (−0.37 1.69)	0.202	0.318
Model 3			
TSI nadir (%)	0.84 (−0.13 1.80)	0.088	0.401
TSI nadir to overshoot difference (%)	−0.77 (−1.98 0.45)	0.208	0.401
Maximum TSI recovery rate (%/s)	−0.15 (−0.46 0.16)	0.324	0.401
Normalized TSI recovery rate (%/mmHg)	−0.02 (−0.05 0.02)	0.401	0.401
TSI baseline to steady-state change (%)	−0.15 (−0.46 0.16)	0.324	0.401
Model 4			
TSI nadir (%)	0.73 (−0.27 1.72)	0.147	0.314
TSI nadir to overshoot difference (%)	−0.86 (−2.06 0.35)	0.157	0.314
Maximum TSI recovery rate (%/s)	−0.17 (−0.47 0.14)	0.282	0.376
Normalized TSI recovery rate (%/mmHg)	-	-	-
TSI baseline to steady-state change (%)	0.45 (−0.84 1.73)	0.484	0.484

Model 1: univariate, Model 2: model 1 + gender, Model 3: model 2 + gender + body mass index + HR baseline, Model 4: model 3 + corresponding SBP variable. HR: heart rate, SBP: systolic blood pressure, TSI: tissue saturation index. * *p* < 0.05.

## Data Availability

The data presented in this study are available on request from the corresponding author.

## Appendix A

**Table A1:** Table 1 expanded with all features extracted and calculation method detailed.

Variable	Units	Description	Calculation Method
SBP baseline	mmHg	Mean SBP 30–60 s before standing	$\frac{1}{n}\;\sum_{t\;=-60}^{-30}{\mathrm{SBP}}_{t}$
DBP baseline	mmHg	Mean DBP 30–60 s before standing	$\frac{1}{n}\;\sum_{t\;=-60}^{-30}{\mathrm{DBP}}_{t}$
HR baseline	bpm	Mean HR 30–60 s before standing	$\frac{1}{n}\;\sum_{t\;=-60}^{-30}{\mathrm{HR}}_{t}$
TSI baseline	%	Mean TSI 30–60 s before standing	$\frac{1}{n}\;\sum_{t\;=-60}^{-30}{\mathrm{TSI}}_{t}$
Δ[O_2_Hb] baseline	µmol/L	Mean Δ[O_2_Hb] 30–60 s before standing	$\frac{1}{n}\;\sum_{t\;=-60}^{-30}\Delta {\left[O_{2}\mathrm{Hb}\right]}_{t}$
Δ[HHb] baseline	µmol/L	Mean Δ[HHb] 30–60 s before standing	$\frac{1}{n}\;\sum_{t\;=-60}^{-30}\Delta {\left[\mathrm{HHb}\right]}_{t}$
SBP nadir	mmHg	Largest trough in SBP after standing (within 30 s)	$\min(\mathrm{local}\;\mathrm{minima}({\mathrm{SBP}}_{t})),$ t=0,…,30
TSI nadir	%	Largest trough in TSI after standing (within 30 s)	$\min(\mathrm{local}\;\mathrm{minima}({\mathrm{TSI}}_{t})),$ t=0,…,30
Δ[O_2_Hb] nadir	µmol/L	Largest trough in Δ[O_2_Hb] after standing (within 30 s)	$\min(\mathrm{local}\;\mathrm{minima}(\Delta \left[O_{2}\mathrm{Hb}{]}_{t}\right)$ , t=0,…,30
Δ[HHb] peak	µmol/L	Largest peak in Δ[HHb] after standing (within 30 s)	$\max(\mathrm{local}\;\mathrm{maxima}\left(\Delta {\left[\mathrm{HHb}\right]}_{t}\right),$ t=0,…,30
SBP overshoot	mmHg	First peak found between 5 s after SBP nadir and 60 s after standing	$\mathrm{first}(\mathrm{local}\;\mathrm{maxima}({\mathrm{SBP}}_{t})),\;$ t= nadir+5,…,60
TSI overshoot	%	First peak found between 2 s after TSI nadir and 60 s after standing	$\mathrm{first}(\mathrm{local}\;\mathrm{maxima}({\mathrm{TSI}}_{t})),\;$ t= nadir+2,…,60
Δ[O_2_Hb] overshoot	µmol/L	First peak found between 2 s after Δ[O_2_Hb] nadir and 60 s after standing	$\mathrm{first}\left(\mathrm{local}\;\mathrm{maxima}\left(\Delta {\left[O_{2}\mathrm{Hb}\right]}_{t}\right)\right),$ t= nadir+2,…,60
Δ[HHb] trough	µmol/L	First trough found between 2 s after Δ[HHb] peak and 60 s after standing	$\mathrm{first}(\mathrm{local}\;\mathrm{minima}\left(\Delta \left[\mathrm{HHb}{]}_{t}\right)\right),\;t=\;\mathrm{nadir}+2,\ldots ,60$
SBP steady-state	mmHg	Mean SBP 60–120 s after standing	$\frac{1}{n}\;\sum_{t\;=60}^{120}{\mathrm{SBP}}_{t}$
TSI steady-state	mmHg	Mean TSI 60–120 s after standing	$\frac{1}{n}\;\sum_{t\;=60}^{120}{\mathrm{TSI}}_{t}$
Δ[O_2_Hb] steady-state	µmol/L	Mean Δ[O_2_Hb] 60–120 s after standing	$\frac{1}{n}\;\sum_{t\;=60}^{120}\Delta {\left[O_{2}\mathrm{Hb}\right]}_{t}$
Δ[HHb] steady-state	µmol/L	Mean Δ[HHb] 60–120 s after standing	$\frac{1}{n}\;\sum_{t\;=60}^{120}\Delta {\left[\mathrm{HHb}\right]}_{t}$
Delta SBP	mmHg	SBP baseline—SBP nadir	SBP_B_ − SBP_N_
Delta TSI	%	TSI baseline—TSI nadir	TSI_B_ − TSI_N_
Delta Δ[O_2_Hb]	µmol/L	Δ[O_2_Hb] baseline—Δ[O_2_Hb] nadir	Δ[O_2_Hb]_B_ − Δ[O_2_Hb]_N_
Delta Δ[HHb]	µmol/L	Δ[HHb] baseline—Δ[HHb]peak	Δ[HHb]_B_ − Δ[HHb]_P_
SBP nadir to overshoot difference	mmHg	SBP overshoot—SBP nadir	SBP_O_ − SBP_N_
TSI nadir to overshoot difference	%	TSI overshoot—TSI nadir	TSI_O_ − TSI_N_
Δ[O_2_Hb] nadir to overshoot difference	µmol/L	Δ[O_2_Hb] overshoot—Δ[O_2_Hb] nadir	Δ[O_2_Hb]_O_ − Δ[O_2_Hb]_N_
Δ[HHb] peak to through difference	µmol/L	Δ[HHb] trough—Δ[HHb] peak	Δ[HHb]_T_ − Δ[HHb]_P_
SBP steady-state change	mmHg	SBP baseline—SBP steady-state	SBP_B_ − SBP_SS_
TSI steady-state change	%	TSI baseline—TSI steady-state	TSI_B_ − TSI_SS_
Δ[O_2_Hb] steady-state change	µmol/L	Δ[O_2_Hb] baseline—Δ[O_2_Hb] steady-state	Δ[O_2_Hb]_B_ − Δ[O_2_Hb]_SS_
Δ[HHb] steady-state change	µmol/L	Δ[HHb] baseline—Δ[HHb] steady-state	Δ[HHb]_B_ − Δ[HHb]_SS_
Maximum SBP recovery rate	mmHg/s	In the range between SPB nadir and SBP overshoot: largest difference between consecutive points/time between points	Max((SBP_i+1_ − SBP_i_)/(1/fs)), i = nadir index,…, overshoot index
Maximum TSI recovery rate	%/s	In the range between TSI nadir and TSI overshoot: largest difference between consecutive points/time between points	Max((TSI_i+1_ − TSI_i_)/(1/fs)), i = nadir index,…, overshoot index
Δ[O_2_Hb] maximum recovery rate	µmol/L/s	In the range between Δ[O_2_Hb] nadir and Δ[O_2_Hb] overshoot: largest difference between consecutive points/time between points	Max((Δ[O_2_Hb]_i+1_ − Δ[O_2_Hb]_i_)/(1/fs)), i = nadir index,…, overshoot index
Δ[HHb] maximum recovery rate	µmol/L/s	In the range between Δ[HHb] nadir and Δ[HHb] overshoot: largest difference between consecutive points/time between points	Max((Δ[HHb] _i+1_ − Δ[HHb] _i_)/(1/fs)), i = nadir index,…, overshoot index
Normalized TSI recovery rate	%/mmHg	Maximum TSI recovery rate/maximum SBP recovery rate	TSI_RR_/SBP_RR_
Normalized Δ[O_2_Hb] recovery rate	µmol/L/mmHg	Δ[O_2_Hb] maximum recovery rate/maximum SBP recovery rate	Δ[O_2_Hb]_RR_/SBP_RR_
Normalized Δ[HHb] recovery rate	µmol/L/mmHg	Δ[HHb] maximum recovery rate/maximum SBP recovery rate	Δ[HHb]_RR_/SBP_RR_

DBP = diastolic blood pressure, Δ[HHb] = concentration of deoxygenated hemoglobin, HR = heart rate, Δ[O_2_Hb] = concentration of oxygenated hemoglobin, SBP = systolic blood pressure, TSI = tissue saturation index.

**Table A2:** Univariate (model 1) and multivariate analyses of the effects of group (patients vs. controls (reference)) on Δ[O_2_Hb] responses to active stand testing.

	**β Coefficient (CI)**	***p*** **-Value**	**FDR** ***p*** **-Value**
Model 1			
Δ[O_2_Hb] nadir (µmol/L)	3.62 (0.05 7.19)	0.047 *	0.235
Δ[O_2_Hb] nadir-overshoot difference (µmol/L)	0.07 (−1.33 1.47)	0.919	0.919
Maximum Δ[O_2_Hb] recovery rate (µmol/L/s)	−0.10 (−0.47 0.26)	0.572	0.715
Normalized Δ[O_2_Hb] recovery rate (µmol/L·mmHg)	−0.02 (−0.06 0.02)	0.292	0.487
Δ[O_2_Hb] baseline to steady-state change (µmol/L)	2.38 (−2.05 6.82)	0.284	0.487
Model 2			
Δ[O_2_Hb] nadir (µmol/L)	2.87 (−0.60 6.35)	0.102	0.432
Δ[O_2_Hb] nadir-overshoot difference (µmol/L)	−0.17 (−1.56 1.23)	0.810	0.810
Maximum Δ[O_2_Hb] recovery rate (µmol/L/s)	−0.14 (−0.52 0.23)	0.441	0.684
Normalized Δ[O_2_Hb] recovery rate (µmol/L·mmHg)	−0.03 (−0.06 0.01)	0.173	0.432
Δ[O_2_Hb] baseline to steady-state change (µmol/L)	1.33 (−3.09 5.74)	0.547	0.684
Model 3			
Δ[O_2_Hb] nadir (µmol/L)	3.24 (−0.67 7.15)	0.101	0.168
Δ[O_2_Hb] nadir-overshoot difference (µmol/L)	−0.68 (−2.13 0.77)	0.346	0.432
Maximum Δ[O_2_Hb] recovery rate (µmol/L/s)	−0.37 (−0.72 −0.01)	0.046 *	0.168
Normalized Δ[O_2_Hb] recovery rate (µmol/L·mmHg)	−0.04 (−0.08 0.00)	0.070	0.168
Δ[O_2_Hb] baseline to steady-state change (µmol/L)	1.77 (−3.27 6.81)	0.481	0.481
Model 4			
Δ[O_2_Hb] nadir (µmol/L)	2.53 (−1.38 6.45)	0.197	0.263
Δ[O_2_Hb] nadir-overshoot difference (µmol/L)	−0.88 (−2.22 0.46)	0.191	0.263
Maximum Δ[O_2_Hb] recovery rate (µmol/L/s)	−0.38 (−0.74 −0.02)	0.042 *	0.168
Normalized Δ[O_2_Hb] recovery rate (µmol/L·mmHg)	-	-	
Δ[O_2_Hb] baseline to steady-state change (µmol/L)	1.54 (−4.02 7.10)	0.577	0.577

Model 1: univariate, Model 2: model 1 + gender Model 3: model 2 + body mass index + HR baseline, Model 4: model 3 + corresponding SBP variable. * *p* < 0.05. HR = heart rate, Δ[O_2_Hb] = concentration of oxygenated hemoglobin, SBP = systolic blood pressure.

**Table A3:** Univariate (model 1) and multivariate analyses of the effects of group (patients vs. controls (reference)) on Δ[HHb] responses to active stand testing.

	β Coefficient (CI)	*p*-Value	FDR *p*-Value
Model 1			
Δ[HHb] peak (µmol/L)	−1.09 (−2.07 −0.12)	0.029 *	0.036 *
Δ[HHb] peak-to-trough difference (µmol/L)	0.93 (0.16 1.71)	0.019 *	0.032 *
Maximum Δ[HHb] recovery rate (µmol/L/s)	0.29 (0.09 0.48)	0.005 **	0.013 *
Normalized Δ[HHb] recovery rate (µmol/L·mmHg)	0.05 (0.02 0.08)	0.002 *	0.010 *
Δ[HHb] baseline to steady-state change (µmol/L)	0.34 (−1.85 2.52)	0.756	0.756
Model 2			
Δ[HHb] peak (µmol/L)	−1.20 (−2.19 −0.21)	0.019 *	0.024 *
Δ[HHb] peak-to-trough difference (µmol/L)	1.02 (0.23 1.805)	0.013 *	0.022 *
Maximum Δ[HHb] recovery rate (µmol/L/s)	0.30 (0.10 0.495)	0.004 **	0.010 *
Normalized Δ[HHb] recovery rate (µmol/L·mmHg)	0.05 (0.03 0.083)	0.001 **	0.005 **
Δ[HHb] baseline to steady-state change (µmol/L)	−0.26 (−2.40 1.876)	0.807	0.807
Model 3			
Δ[HHb] peak (µmol/L)	−0.85 (−1.94 0.24)	0.123	0.154
Δ[HHb] peak-to-trough difference (µmol/L)	1.24 (0.36 2.12)	0.007 **	0.012 *
Maximum Δ[HHb] recovery rate (µmol/L/s)	0.38 (0.18 0.60)	0.001 **	0.002 **
Normalized Δ[HHb] recovery rate (µmol/L·mmHg)	0.06 (0.03 0.09)	0.001 **	0.002 **
Δ[HHb] baseline to steady-state change (µmol/L)	−0.17 (−2.6 2.27)	0.891	0.891
Model 4			
Δ[HHb] peak (µmol/L)	−0.93 (−2.06 0.21)	0.106	0.141
Δ[HHb] peak-to-trough difference (µmol/L)	1.21 (0.32 2.11)	0.009 **	0.018 *
Maximum Δ[HHb] recovery rate (µmol/L/s)	0.39 (0.17 0.60)	0.001 **	0.004 **
Normalized Δ[HHb] recovery rate (µmol/L·mmHg)	-	-	-
Δ[HHb] baseline to steady-state change (µmol/L)	0.09 (−2.59 2.76)	0.949	0.949

Model 1: univariate, Model 2: model 1 + gender Model 3: model 2 + body mass index + HR baseline, Model 4: model 3 + corresponding SBP variable. * *p* < 0.05, ** *p* < 0.01. HR = heart rate, Δ[HHb] = concentration of deoxygenated hemoglobin, SBP = systolic blood pressure.

**Table A4:** Univariate (model 1) and multivariate analyses of the effects of group (patients vs. controls (reference)) on SBP responses to active stand testing.

	β Coefficient (CI)	*p*-Value	FDR Corrected *p*-Value
Model 1			
Delta SBP nadir (mmHg)	5.67 (−4.13 15.47)	0.249	0.249
Delta SBP nadir to overshoot (mmHg)	5.51 (−3.19 14.20)	0.207	0.249
Maximum SBP recovery rate (mmHg/s)	1.22 (−0.70 3.14)	0.205	0.249
Delta SBP steady-state (mmHg)	10.80 (3.42 18.17)	0.005 *	0.020 *
Model 2			
Delta SBP nadir (mmHg)	3.36 (−6.40 13.12)	0.490	0.490
Delta SBP nadir to overshoot (mmHg)	7.63 (−1.00 16.25)	0.081	0.127
Maximum SBP recovery rate (mmHg/s)	1.63 (−0.30 3.56)	0.095	0.127
Delta SBP steady-state (mmHg)	9.34 (1.88 16.80)	0.016	0.064
Model 3			
Delta SBP nadir (mmHg)	4.99 (−5.52 15.49)	0.342	0.438
Delta SBP nadir to overshoot (mmHg)	4.15 (−4.30 12.61)	0.326	0.438
Maximum SBP recovery rate (mmHg/s)	0.60 (−0.95 2.15)	0.438	0.438
Delta SBP steady-state (mmHg)	8.37 (0.44 16.30)	0.039 *	0.156

Model 1: univariate; Model 2: model 1 + gender; Model 3: model 2 + body mass index + HR baseline. * *p* < 0.05. HR = heart rate, SBP = systolic blood pressure.

**Table A5:** Sensitivity analysis to lying duration and speed of standing.

	β Coefficient (CI)	*p*-Value	FDR Corrected *p*-Value
Delta TSI nadir (%)	1.00 (0.05 1.94)	0.039 *	0.195
Delta TSI nadir to overshoot (%)	−0.95 (−2.17 0.27)	0.121	0.258
Maximum TSI recovery rate (%/s)	−0.20 (−0.50 0.11)	0.203	0.258
Normalized TSI recovery rate (%/mmHg)	−0.02 (−0.06 0.02)	0.283	0.283
Delta TSI baseline to steady-state (%)	0.76 (−0.44 1.95)	0.206	0.258

Model: patients vs. healthy + gender + body mass index + HR baseline + lying duration + speed of standing + SBP corresponding feature (for all features except for the normalized TSI recovery rate). HR = heart rate, SBP = systolic blood pressure, TSI = tissue saturation index. * *p* < 0.05.

**Table A6:** Univariate (model 1) and multivariate analyses of the effects of group (patients vs. controls (reference)) on TSI responses to active stand testing using averaged rates for TSI recovery rate and normalized TSI recovery rate.

	β Coefficient (CI)	*p*-Value	FDR Corrected *p*-Value
Model 1			
TSI nadir (%)	0.93 (0.12 1.74)	0.026 *	0.130
TSI nadir to overshoot difference (%)	−0.53 (−1.55 0.49)	0.300	0.375
Average TSI recovery rate (%/s)	−0.04 (−0.19 0.11)	0.587	0.587
Normalized TSI recovery rate (%/mmHg)	−0.02 (−0.06 0.02)	0.285	0.375
TSI baseline to steady-state change (%)	0.72 (−0.27 1.71)	0.148	0.370
Model 2			
TSI nadir (%)	0.84 (0.00 1.68)	0.050	0.250
TSI nadir to overshoot difference (%)	−0.62 (−1.68 0.44)	0.242	0.302
Average TSI recovery rate (%/s)	−0.05 (−0.21 0.10)	0.483	0.483
Normalized TSI recovery rate (%/mmHg)	−0.03 (−0.07 0.01)	0.116	0.290
TSI baseline to steady-state change (%)	0.66 (−0.37 1.69)	0.202	0.302
Model 3			
TSI nadir (%)	0.84 (−0.13 1.80)	0.088	0.240
TSI nadir to overshoot difference (%)	−0.77 (−1.98 0.45)	0.208	0.260
Average TSI recovery rate (%/s)	−0.12 (−0.284 0.05)	0.167	0.260
Normalized TSI recovery rate (%/mmHg)	−0.04 (−0.08 0.01)	0.096	0.240
TSI baseline to steady-state change (%)	−0.15 (−0.46 0.16)	0.324	0.324
Model 4			
TSI nadir (%)	0.73 (−0.27 1.72)	0.147	0.209
TSI nadir to overshoot difference (%)	−0.86 (−2.06 0.35)	0.157	0.209
Average TSI recovery rate (%/s)	−0.13 (−0.30 0.04)	0.132	0.209
Normalized TSI recovery rate (%/mmHg)	-	-	-
TSI baseline to steady-state change (%)	0.45 (−0.84 1.73)	0.484	0.484

Model 1: univariate, Model 2: model 1 + gender, Model 3: model 2 + gender + body mass index + SBP baseline, Model 4: model 3 + corresponding SBP variable. HR = heart rate, SBP = systolic blood pressure, TSI = tissue saturation index. TSI recovery rate is calculated as: (TSI overshoot − TSI nadir)/(TSI overshoot time − TSI nadir time). Normalized TSI recovery rate is calculated as: ((TSI overshoot − TSI nadir)/(TSI overshoot time − TSI nadir time))/((SBP overshoot − SBP nadir)/(SBP overshoot time − SBP nadir time)). HR = heart rate, SBP = systolic blood pressure, TSI = tissue saturation index. * *p* < 0.05.

**Table A7:** Univariate (model 1) and multivariate analyses of the effects of group (patients vs. controls(reference)) on TSI responses to active stand testing using a subcohort of patients matched by age and gender to the controls.

	β Coefficient (CI)	*p*-Value	FDR Corrected *p*-Value
Model 1			
Delta TSI (%)	0.81 (−0.22 1.846)	0.117	0.454
TSI nadir-overshoot difference (%)	−0.61 (−1.95 0.742)	0.363	0.454
TSI recovery rate (%/s)	−0.00 (−0.36 0.350)	0.981	0.981
Normalized TSI recovery rate (%/mmHg)	−0.02 (−0.06 0.020)	0.343	0.454
TSI baseline to steady-state change (%)	0.54 (−0.58 1.655)	0.333	0.454
Model 3			
Delta TSI (%)	0.73 (−0.52 1.98)	0.238	0.697
TSI nadir-overshoot difference (%)	−0.66 (−2.29 0.97)	0.411	0.697
TSI recovery rate (%/s)	−0.08 (−0.50 0.34)	0.697	0.697
Normalized TSI recovery rate (%/mmHg)	−0.01 (−0.05 0.03)	0.647	0.697
TSI baseline to steady-state change (%)	0.51 (−0.86 1.88)	0.445	0.697
Model 4			
Delta TSI (%)	0.68 (−0.62 1.98)	0.287	0.614
TSI nadir-overshoot difference (%)	−0.84 (−2.50 0.83)	0.307	0.614
TSI recovery rate (%/s)	−0.14 (−0.57 0.29)	0.498	0.663
Normalized TSI recovery rate (%/mmHg)	-	-	-
TSI baseline to steady-state change (%)	0.32 (−1.19 1.83)	0.663	0.663

Model 1: univariate, Model 3: model 1 + body mass index + HR baseline, Model 4: model 3 + corresponding SBP variable. SBP = systolic blood pressure, TSI = tissue saturation index.

**Table A8:** Univariate (model 1) and multivariate analyses of the effects of group (patients vs. controls (reference)) on SBP responses to active stand testing using a subcohort of patients matched by gender to the controls.

	β Coefficient (CI)	*p*-Value	FDR Corrected *p*-Value
Model 1			
Delta SBP (%)	1.25 (−11.92 14.41)	0.847	0.847
SBP nadir-overshoot difference (%)	10.31 (−0.73 21.35)	0.066	0.088
SBP recovery rate (%/s)	2.53 (0.14 4.92)	0.039 *	0.088
SBP baseline to steady-state change (%)	9.05 (−0.48 18.58)	0.062	0.088
Model 3			
Delta SBP (%)	2.87 (−11.56 17.30)	0.684	0.684
SBP nadir-overshoot difference (%)	6.65 (−4.44 17.75)	0.227	0.303
SBP recovery rate (%/s)	1.36 (−0.64 3.36)	0.173	0.303
SBP baseline to steady-state change (%)	7.83 (−2.56 18.21)	0.132	0.303

Model 1: univariate, Model 3: model 1 + body mass index + HR baseline. * *p* < 0.05. SBP = systolic blood pressure, TSI = tissue saturation index.
